# Dihydroxyadenine crystalline nephropathy: an under-recognized cause of rapidly progressive renal failure. A Nephrology picture

**DOI:** 10.1007/s40620-024-02143-y

**Published:** 2024-12-19

**Authors:** Sherif Mansour, Wesam Ismail, Haidy Mohammed Zakaria

**Affiliations:** 1 Nephrology Department, Alrowad Private Hospital, Menoufia, 32951 Egypt; 2 Pathology Department, Faculty of Medicine, Beni-Suef University, Beni Suef, Egypt; 3 Department of Clinical Research and Health Development, Menoufia Directorate of Health Affairs, Ministry of Health and Population, 32511 Shebin El-Kom, Menoufia, Egypt

**Keywords:** Crystalline nephropathy, Dihydroxyadenine, Renal failure

Impaired kidney function was discovered in a 57-year-old man seeking medical advice for elevated blood pressure. Urine output had decreased in the 3 days before referral. There was no past history of renal disease or nephrotoxic drug intake. The patient denied a family history of kidney diseases.

At presentation, examination revealed anasarca and elevated blood pressure (150/100 mmHg), but no hepatomegaly or splenomegaly. Heart bruits were normal, and chest ascultation was clear.

Laboratory investigations revealed serum creatinine of 2.3 mg/dl and urea of 98 mg/dL. Serum calcium, phosphorus, and magnesium were 9.4, 3.6, and 2.1 mg/dl, respectively. Serum sodium concentration was 115 mmol/l, and serum potassium was 3.1 mmol/l;  hemoglobin was 12.3 g/dl, total leukocyte count 10.6 × 10^9^/L, and platelet count 217 × 10^9^/L. Aspartate aminotransferase was 130 IU/L, alanine aminotransferase was 136 IU/L, and serum albumin was 3.8 g/dl. HbA1C was 6.3%. Urinalysis showed urate crystals (+ +), 8–10 red blood cells, and 20–25 pus cells/high power field.

Renal ultrasounds revealed kidneys of normal size, bilateral hyperechogenicity and no kidney stones. Eye fundus examination was unremarkable.

Viral markers revealed HCV-Ab positivity, while ANCA C and P, ANA, and rheumatoid factor were absent.

The patient received antihypertensive and intravenous diuretic therapy. Edema subsided but serum creatinine gradually increased to 8.3 mg/dl within one month, associated with the appearance of uremic symptoms.

Due to the rapid deterioration of kidney function, a renal biopsy was performed and revealed numerous brown crystals in the tubular lumen. Tubules showed acute tubular injury with dilatation, and flattening of the epithelium, tubular atrophy and small vessel hyalinosis. Hyaline casts were also seen. The interstitial tissue contained chronic inflammatory cells, mainly lymphocytes, with edema and areas of fibrosis (Figs. 1 & 2).

**Fig. 1:**
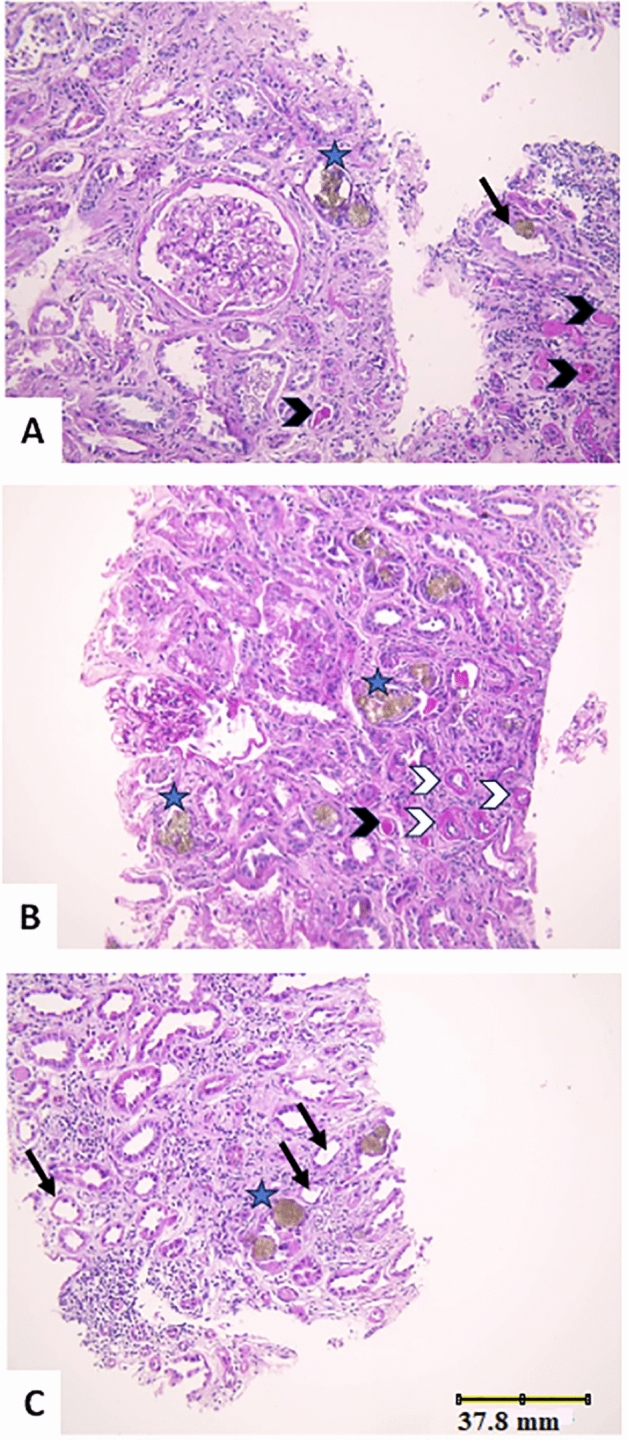
Histopathological assessment of the renal biopsy, magnification power (× 200): **A**, **B** and **C**: H&E stained images showing two unremarkable glomeruli (in **A** and **B**). Some tubules showing reddish-brown crystals within the tubular lumina (blue Asterisk) (images **A**, **B** &**C**). The tubules show acute tubular injury (black arrow) (images A&C). Other tubules (B) show tubular atrophy with irregular outlines and hyalinosis of the small vessel walls (white arrowhead). Hyaline casts are also seen (black arrowhead) (images**A** &**B**). The interstitial tissue with chronic inflammatory cells mainly contains lymphocytes (images A&C)

**Fig. 2:**
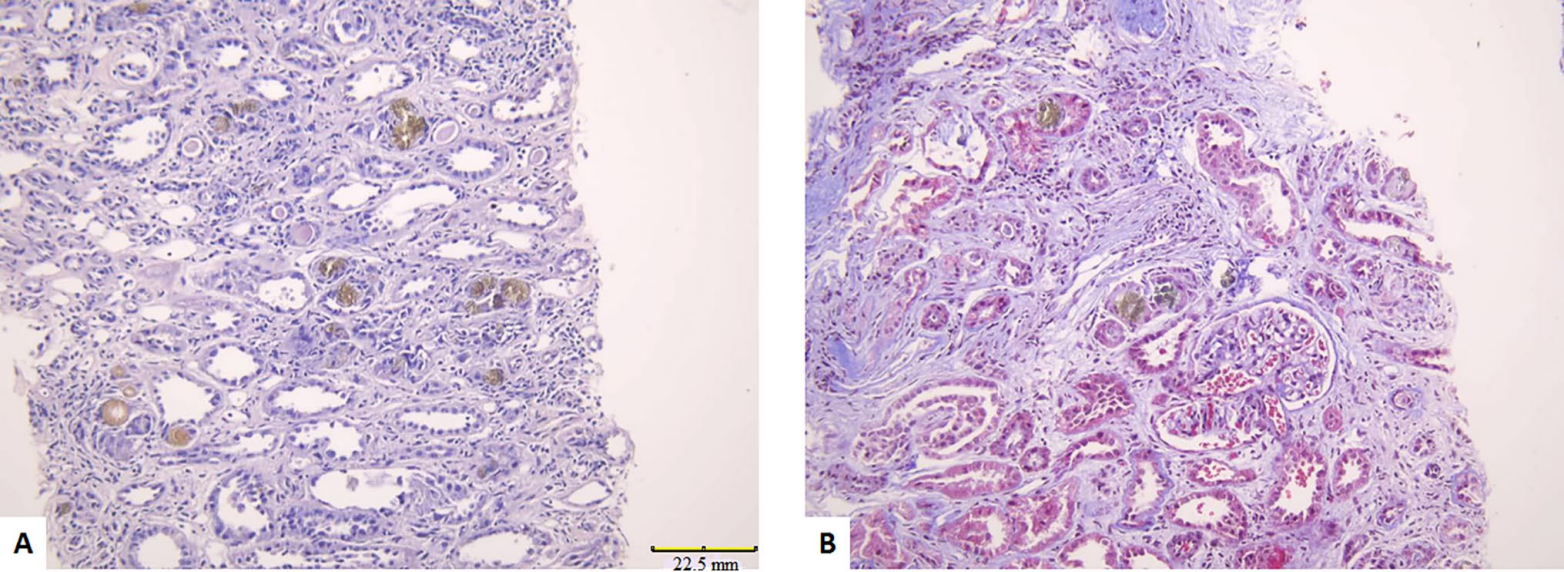
Masson trichrome stain showing edema of the interstitial tissue with areas of fibrosis, magnification (× 200)

A diagnosis of dihydroxyadenine crystal nephropathy was made and the patient was started on the xanthine oxidase inhibitor febuoxsat (80 mg/day) and prescribed a low-salt, low-fat, high fluid diet. His symptoms rapidly improved. Serum creatinine decreased to 2 mg/dl after 6 weeks of starting of the xanthine oxidase inhibitor.

Crystalline nephropathies are a specific form of kidney disease with the characteristic histologic finding of crystal deposition in the tubular lumen, which promotes kidney injury through tubular obstruction and both direct and indirect cytotoxicity. Phagocytosed crystals destabilize lysosomes, which release their contents, inducing cellular stress and autophagic cell death (1).Timely recognition of crystals in urine or on biopsy is important. The non-purine XDH inhibitor febuxostat is a highly effective therapeutic option (2).

## Supplementary Information

Below is the link to the electronic supplementary material.

Supplementary file1 (DOCX 14 KB)

## Data Availability

All data generated or analyzed during this study are included in this published article.
