# Supplementary material for: Dihydroxyadenine crystalline nephropathy: an under-recognized cause of rapidly progressive renal failure. A Nephrology picture
Source: J Nephrol. 2024 Dec 19;38(1):263–5. doi: 10.1007/s40620-024-02143-y (PMC11903589; doi:10.1007/s40620-024-02143-y)
Supplement: Supplementary file 1 — Supplementary file1 (DOCX 14 KB) [file 40620_2024_2143_MOESM1_ESM.docx]

**Response to reviewers' comments**

Dear my professors

Thanks for reconsidering our manuscript and for the reviewers comments which we took seriously and did our best to conform to.

**Comment 1:** Apparently the hyponatremia is no longer secondary to the hypovolemia but to the hypervolemia because the patient was overloaded with fluids. However, this is not written in the case report and it is not clear why it was infused given that the patient was also hypertensive. I would advise the authors to be more clear and to explain in the case report the therapy carried out on the patient and the reason for this therapy if any.

**Response 1:** yes, I missed to report an important point in the case presentation which made the confusion. The patient presented to us with generalized anasarca due to a period of oliguria preceded his admission by 3 [days, this was the main cause of hypervolemia at](http://days.at) that time investigation revealed hyponatremia Which improved after giving the patient iv diuretic therapy to restoring the normal urine volume with subsiding of the generalized edema at the first presentation of the patient.

I made the presentation more clear by adding the history of anasarca and added the management in the history.
